# Enhancing smoked meat (Se’i sapi) quality: The impact of adding essential oils of cinnamon and lime leaf during room temperature storage

**DOI:** 10.5455/javar.2025.l869

**Published:** 2025-03-23

**Authors:** Restu Ratih Kinasih, Safna Fauziah, Usamah Abdi Kafa, Dita Aviana Devi, Pradita Iustitia Sitaresmi, Edi Suryanto, Andi Febrisiantosa, Annisa Kusumaningrum, Aldicky Faisal Amri, Eki Prilla Sulistyono, Bayu Murti Dewandaru, Asep Nurhikmat, Endy Triyannanto

**Affiliations:** 1 Department of Animal Products Technology, Faculty of Animal Science, Universitas Gadjah Mada, Yogyakarta, Indonesia; 2 Graduate School of Faculty of Animal Science, Universitas Gadjah Mada, Yogyakarta, Indonesia; 3 Research Center for Animal Husbandry, National Research and Innovation Agency, Cibinong Science Center, Jalan Raya Jakarta-Bogor, Bogor, Indonesia.; 4 Research Center for Food Technology and Processing, National Research and Innovation Agency of Indonesia, Gunungkidul, Indonesia

**Keywords:** Antioxidant, essential oils, meat preservation, meat product

## Abstract

**Objective:**

This study examines the effects of lime leaf and cinnamon essential oils (EOs) at different concentrations and storage durations on the quality of Se’i under ambient conditions.

**Materials and Methods:**

We used a two-factor completely randomized design with three replications. The first factor involved EO concentrations of 0.1%, 0.3%, and 0.5%, while the second factor was storage duration at 0, 7, 14, and 21 days. Key parameters evaluated included pH, tenderness, thiobarbituric acid values, meat color, and sensory analysis.

**Results:**

The results indicated that adding EOs effectively preserved the smoked meat, with improved quality parameters compared to the control group.

**Conclusion:**

Adding 0.5% lime leaf or cinnamon essential oil to Se'i made it taste and look much better, showing that it could be used as a natural way to keep smoked meats fresh.

## Introduction

The escalating demand for beef products is on a steady rise, triggering a parallel surge in innovations for meat preservation. Preserving meat demands meticulous methods to shield it from damage, given its inherently perishable nature and its propensity to serve as an ideal medium for microbial growth, resulting in a brief shelf life [1]. Indonesia exhibits Indigenous expertise in augmenting the shelf life of meat products through the production of se’i meat, presenting promising avenues for future development [2]. Se’i meat, a processed form of smoked meat indigenous to East Nusa Tenggara, has gained significant popularity and is currently in high demand among the Indonesian population. Se’i usually comes from beef, pork, and deer to obtain meat with a distinctive aroma and taste. The preparation of se’i combines smoking and curing with table salt (NaCl), enhancing flavor, stabilizing color, and creating its distinctive aroma. These methods effectively preserve the meat, extending its shelf life without refrigeration, making it ideal for regions with limited access to modern preservation techniques [3].

The high public interest in Se’i sapi is due to its nutritional value and high protein content, which is 30%–32%, and fat, which is 0.81%–0.92%, but compared to other processed beef products such as beef jerky and shredded meat, Se’i meat has higher water content, which is 60%. The nutrient contents, especially the water concentration in sei, products, remain quite high, rendering these products quite fragile to product deterioration [3]. Water activity is a critical factor in food preservation, as it plays a significant role in microbial growth and chemical reactions. So that makes se`i have a low shelf life of only 3–7 days. By lowering water activity, the growth of spoilage organisms and pathogens can be inhibited. In the case of se'i sapi, its high water content (60%) makes it especially susceptible to microbial contamination. This heightened vulnerability increases the potential for damage to this type of processed meat, making the use of preservatives essential to maintain its quality.

This deterioration of meat products alters various nutritional properties as well as the physical condition of the product, such as structure, pH, and aroma [4]. The other major deterioration factor of meat products is the process of oxidation during the storage period of the processed products [5]. These microbial and oxidation factors need to be considered as preservation controls for processed meat products [3]. Microbial contamination is a major concern for meat products, especially those with high moisture content. The study mentions the susceptibility of se'i sapi to microorganisms, but it does not delve into specific microbiological control measures. Implementing good manufacturing practices, such as maintaining hygiene during processing, proper storage conditions, and using preservatives, can help control microbial growth. Additionally, regular microbiological testing during storage could provide insights into the safety and quality of the product over time. The low shelf life of se’i meat can be increased by using vacuum packaging [6] and adding several types of essential oils (EOs) as sources of antioxidants [7] to prevent and slow down the oxidation process.

Plants release volatile organic compounds in response to physiological stress, environmental factors, and pathogen attacks, which are referred to as EOs [8]. These oils exhibit natural antibacterial properties and combat free radicals. Cinnamon and lime leaf EOs are recognized for their diverse benefits, particularly as sources of antioxidants and antibacterial properties, and their role in enhancing the flavor of processed food products, which can also serve as agents for the preservation of processed meat products [9–14]. EOs, derived from natural sources, are predominantly classified as Generally Recognized as Safe (GRAS) due to their low toxicity levels, making them more suitable for consumption. Their application is increasingly preferred over artificial preservatives [3].

Enhancing the shelf life of meat products is crucial for maintaining quality and ensuring economic viability. Comprehending the extrinsic and intrinsic factors influencing meat products is essential for optimizing shelf life. Cinnamon and lime leaf EOs may serve as natural preservatives and enhance the aroma of meat products. This research aims to identify the effects and potency of adding cinnamon and lime leaf EOs, as well as the impact of storage time factors, on the quality of vacuum-packed beef Se'i at room temperature. The study seeks to extend the product's shelf life and estimate the optimal shelf life of beef Se'i using the Arrhenius model, which is anticipated to serve as a reference for the product's consumption limits.

## Materials and Methods

### Ethical approval

This study does not involve any human participants, animal subjects, or sensitive personal data. Therefore, it does not require ethical clearance from an institutional review board or ethics committee.

### Liquid smoke preparation on beef Se’i production

The production of liquid smoke to make beef se’i from Kesambi wood involves several stages: preparation, pyrolysis, distillation, and purification. The process begins with cutting Kesambi wood into pieces approximately 2 cm in size or smaller and drying them thoroughly. The dried wood is then placed in a pyrolysis tube, filling only half the tube to ensure even combustion, and heated indirectly at 400°C–450°C for 8 h. During pyrolysis, smoke is generated within 15–20 min and channeled through a pipe into a condenser cooled with running water, condensing the smoke into a liquid form. Once the pyrolysis stops emitting smoke, the liquid smoke collected is dark and tar-rich. This crude liquid is left to settle for one week to allow tar to separate and is then filtered using active zeolite and filter paper to produce a cleaner product. The final liquid smoke product is a brownish liquid with a strong acidic aroma, demonstrating its utility and quality for various applications.

### Meat preparation and processing se`i sapi

Essential oil solutions were prepared by homogenizing the oils in a total volume of 500 ml for each treatment, following the concentration of the treatment that will be given, ensuring even distribution. The essential oil solutions containing cinnamon (C) and lime leaf (L) EOs were prepared at concentrations of 0.1% m/v (C1; L1), 0.3% m/v (C2; L2), and 0.5% m/v (C3; L3). The marination process involved soaking the fresh meat samples in these solutions for 2 h at 4°C, followed by storage in sterile trays wrapped with air-permeable film. The solution was homogenized at 10,000 rpm for 5 min (T18 digital ultra turrax, IKA, Belgium).

The dimensions and weight of the meat samples used in this study were meticulously measured to ensure consistency. Each sample had an average length of 10 cm, a width of 1.5 cm, and a thickness of 0.5 cm, with an average weight of approximately 15 gm per piece. After preparation, the meat samples were immersed in the marinade, stored in sterile trays wrapped with air-permeable polyethylene film, and refrigerated at 4°C for 2 h to ensure adequate marination. The marinated meat samples were drained and placed in the oven at 140°C for 80 min, following the process of making beef se`i [3]. After that, the meat was vacuum-packed in polypropylene (PP) plastic and kept at room temperature, which was checked before the lab work to be between 27°C and 30°C, which is the same as the temperature at which things usually are in tropical areas.

### pH value

Ten grams of meat samples were chopped using a meat chopper to ensure consistent sizes and transferred into 40 ml of distilled water. The mixture was homogenized using a homogenizer at 10,000 rpm for 60 sec (T18 digital ultra turrax, IKA, Belgium). The pH values were measured using a pH meter (Orion Star A111 Benchtop, Thermo Fisher Scientific Inc., Singapore) equipped with an electrode. Measurements were performed in triplicate for each treatment to ensure accuracy [16].

### Tenderness

Samples of smoked meat (se’i sapi), prepared to a thickness of 0.5 cm, a width of 1.5 cm, and a length of 1.5 cm, were placed on the Warner–Bratzler shear force instrument to measure tenderness parameters. The samples were cut perpendicular to the muscle fiber orientation, and the shear force was measured at a crosshead speed of 2 mm/s, following the method described [3].

### TBA analysis

Thiobarbituric acid (TBA) values were carried out according to the previous procedure [17]. 10 gm of meat samples were blended into 50 ml of aquades and then poured into a distillation flask of 250 ml with the addition of 47.5 ml of aquadest and 2.5 ml of HCl 4 M. After that, the distillation flask was installed on the distillation apparatus and heated until 50 ml of distillate was collected. 5 ml of distillate was mixed up with 5 ml of TBA reagent (0.02 M TBA solution in 90% of glacial acetic acid). Furthermore, the glass containing the solution was heated in boiling water for 35 min and cooled down by using flowing water. Afterward, absorbance was measured with a wavelength of 528 nm. TBA values were conducted once a week for 21 days.

### Meat color analysis

Color was assessed by a Minolta^®^ CR-400 colorimeter (Konica Minolta Sensing Inc., Japan). The CIE system was utilized, and the parameters of lightness (L*), redness (a*), and yellowness (b*) were used to objectively define color [3]. The color determination was performed for each group on 0, 7, 14, and 21 days of storage on se’i sapi samples at room temperature under vacuum packaging.

### Sensory evaluation

Sensory evaluation is a scientific method to measure and analyze the sensory characteristics of food products, such as meat, as perceived by human senses (appearance, color, aroma, texture, and taste). Panelists, typically trained or semi-trained individuals, assess these attributes and provide subjective feedback based on their preferences. A 9-point hedonic scale is commonly used, where panelists rate their liking of specific attributes from 1 (“dislike extremely”) to 9 (“like extremely”). The hedonic scale values are converted into numerical data for statistical analysis. For this study, coded samples of smoked meat (se’i sapi) were presented to 7 trained panelists. The panelists evaluated the samples based on physical appearance, color, aroma, texture, and taste to determine the most preferred formulation among the treatments [35].

### Statistical analysis

The data were analyzed as a completely randomized design using a 2-way ANOVA concerning the kind of dosage EOs (Cinnamons (0.1%; 0.3%; 0.5%) and Lime leaf extract (0.1%; 0.3%; 0.5%) and time its preserve (0, 7, 14, and 21 d) as a factorial design, according to the following linear model: Y_ij_ = μ + A_i_ + B_j_ + (AB)_ij_ + e_ij_, where Y_ij_ = value of trait (the dependent variable); μ = overall mean; A_j_ = effect of kind of dosage EOs; B_j_ = effect of time storage of se’i; (AB) = interaction, and e_ij_ = random observation error, using SPSS ver 25 software. The statistical significance of the differences between the averages of the groups was calculated using Tukey's test and was at a level of *p* ≤ 0.05. The tables present the average values and their standard deviations.

## Results and Discussion

### pH value

The pH values of smoked meat (se’i sapi) samples treated with cinnamon bark oil and lime leaf oil are summarized in Table 1. For the cinnamon bark oil-treated samples, the pH values were as follows: C1 showed pH levels of 5.55, 4.56, 4.49, and 4.55; C2 exhibited pH levels of 5.55, 5.58, 4.59, and 4.55; and C3 recorded pH values of 5.31, 5.37, 5.02, and 5.38 on the respective storage days. Similarly, for the lime leaf oil-treated samples, the pH values were: L1 with 5.61, 5.37, 4.69, and 4.43; L2 with 5.52, 5.51, 4.59, and 5.17; and L3 with 5.41, 4.53, 5.02, and 4.56 across the corresponding storage periods. These variations in pH highlight the influence of EOs on the acidification of smoked meat over time. Compared to the control, all treatments better preserved the meat, maintaining the pH at less acidic levels until day 21 (*p* < 0.05). While pH values tended to decrease during storage, they remained within the acidic range. Various factors influence meat pH, including its protein structure and water-binding capacity. According to a previous study, protein degradation by microorganisms can increase the pH due to the production of alkaline compounds [18]. This aligns with another study that indicates pH increases as beef is stored for extended periods [19].

The se’i sapi samples treated with both cinnamon bark oil and lime leaf oil exhibited fluctuating pH values. Among the treatments, C3 (0.5% cinnamon EOs) and L2 (0.3% lime leaf EOs) maintained pH levels on day 21 better than the other treatments. This result is attributed to the acidic nature of the oils, as lime leaf oil contains citric acid, which contributes to its acidic pH [15]. A rise in pH indicates declining beef quality because higher pH levels allow bacteria to degrade the meat further [20]. However, after a certain point, the pH decreases due to the activity of lactic acid bacteria.

**Figure 1.**
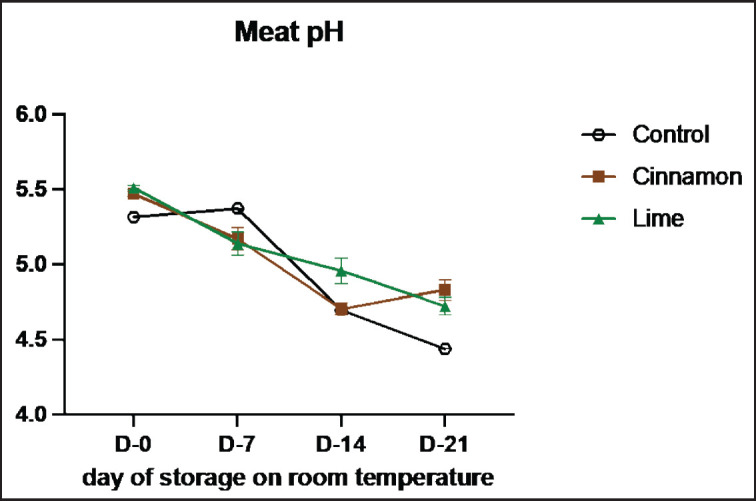
Average results of the addition of Cinnamons and Lime leaf EOs on the change in pH values during 21 days at room temperature.

Samples treated with cinnamon bark oil and lime leaf oil exhibited lower pH reductions compared to the control sample (Fig. 1). The pH reduction in se’i sapi during storage is influenced by several mechanisms. First, microbial activity produces acidic metabolites, lowering the pH. Second, protein degradation by autolytic enzymes, such as cathepsins and calpains, releases amino acids and other acidic products, further reducing the pH. Third, protein denaturation due to prolonged storage increases the concentration of acidic products, accelerating the pH decline [16]. Additionally, microbial contaminants degrade glucose, lactic acid, and some amino acids into alkaline metabolites, which can raise the pH while exerting an antimicrobial effect. Compounds like cinnamaldehyde in cinnamon bark [21] and limonene in lime leaf [22] inhibit microbial growth, thereby preventing the formation of alkaline metabolites. The slower pH reduction in the control samples was attributed to higher levels of alkaline metabolites, whereas the treatment samples exhibited slightly lower levels of these metabolites due to the presence of antimicrobial compounds [23].

### Tenderness

The tenderness values of smoked meat (Se’i sapi) samples are presented in Table 2. The tenderness value of the control sample (C0) was 5.50, 8.14, 2.46, and 6.38 on days 0, 7, 14, and 21 of storage, respectively. When cinnamon bark oil was used, the tenderness of smoked meat (Se’i sapi) for C1 was 6.30, 10.92, 10.24, and 8.72; for C2, the tenderness was 6.10, 7.14, 8.88, and 6.78; and for C3, the tenderness was 7.48, 7.46, 8.06, and 9.42 on the corresponding storage days. When lime leaf oil was used, the tenderness of smoked meat (Se’i sapi) for L1 was 6.68, 9.50, 7.42, and 5.84; for L2, the tenderness values were 7.66, 5.18, 8.62, and 5.94; and for L3, the tenderness was 6.94, 6.04, 6.60, and 9.00 on the corresponding storage days. Meat tenderness was assessed based on the difference in myofibrillar protein and connective tissue, particularly collagen [24].

**Figure 2.**
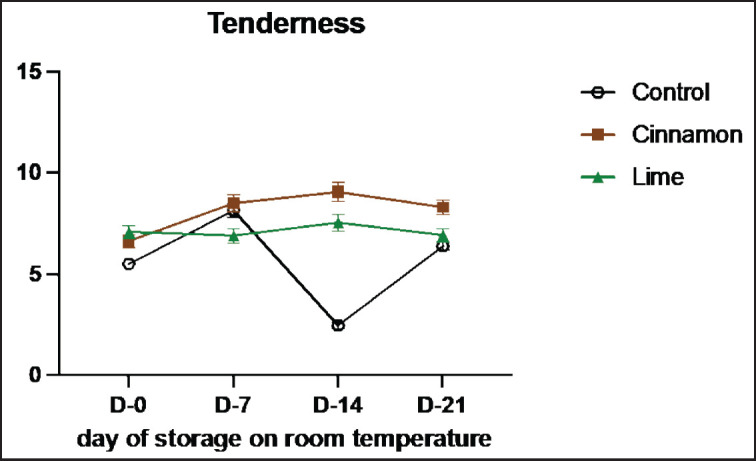
Average results of the addition of Cinnamons and Lime leaf EOs on the change in tenderness values during 21 days at room temperature.

Table 2 and Figure 2 indicated that tenderness was no different between treatments (*p *> 0.05). This could be due to the fact that the amount of EOs added to smoked meat (Se’i sapi) samples was not enough to make any difference. In this research, the addition of both cinnamon and lime leaf oils did not impact the tenderness of smoked meat (Se’i sapi) but showed a bit lower than the control samples might be because all samples passed through the same heating, and the action of phenolic compounds did not affect the texture at the early stage after the process. This result aligns with a previous study that reported that marinade solution applied with essential oil (0.8%) and marinated juice can increase meat tenderness by reducing shear force in pork loin [25].

**Table 1.** Mean pH values of untreated and treated on smoked meat (Se’i sapi) samples during storage period at room temperature (Mean ± SE).

Treatment	Days			
	0	7	14	21
Control	5.31 ± 0.01^a^	5.37 ± 0.02^c^	4.69 ± 0.02^d^	4.44 ± 0.00^a^
C 1 (0.1%)	5.55 ± 0.01^d^	4.56 ± 0.01^b^	4.49 ± 0.01^a^	4.55 ± 0.01^b^
C 2 (0.3 %)	5.55 ± 0.00^d^	5.58 ± 0.02^e^	4.59 ± 0.01^c^	4.55 ± 0.01^b^
C 3 (0.5%)	5.31 ± 0.01^a^	5.37 ± 0.01^c^	5.02 ± 0.01^e^	5.38 ± 0.39^d^
L 1 (0.1%)	5.61 ± 0.01^e^	5.37 ± 0.02^c^	4.69 ± 0.02^d^	4.43 ± 0.01^a^
L 2 (0.3%)	5.52 ± 0.03^c^	5.51 ± 0.02^d^	4.59 ± 0.01^f^	5.17 ± 0.01^c^
L 3 (0.5%)	5.41 ± 0.02^b^	4.53 ± 0.00^a^	5.02 ± 0.01b	4.56 ± 0.02^b^

Results are expressed as mean ± SD.

^abcd^ Significantly different in same column (additional EOs effect) (*p *< 0.05)

**Table 2.** Mean Tenderness values of untreated and treated on smoked meat (Se’i sapi) samples during storage period at room temperature (Mean ± SE).

Treatment	Days			
	0	7	14	21
Control	5.50 ± 1.11	8.14 ± 2.0.06^abc^	2.46 ± 0.95^a^	6.38 ± 1.09^ab^
C 1 (0.1%)	6.30 ± 1.38	10.92 ± 2.06^c^	10.24 ± 3.00^b^	8.72 ± 1.59^ab^
C 2 (0.3 %)	6.10 ± 2.18	7.14 ± 1.41^ab^	8.88 ± 3.46^b^	6.78 ± 2.32^ab^
C 3 (0.5%)	7.48 ± 2.21	7.46 ± 1.79^ab^	8.06 ± 1.97^b^	9.42 ± 2.04^b^
L 1 (0.1%)	6.68 ± 2.42	9.50 ± 0.55^bc^	7.42 ± 1.19^b^	5.84 ± 1.47^a^
L 2 (0.3%)	7.66 ± 1.83	5.18 ± 1.36^a^	8.62 ± 4.09^b^	5.94 ± 1.47a
L 3 (0.5%)	6.94 ± 1.64	6.04 ± 1.05^a^	6.60 ± 0.83^ab^	9.00 ± 1.04^ab^

Results are expressed as mean ± SD.

^abcd^ Significantly different in same column (additional EOs effect) (*p* < 0.05)

The addition of EOs in certain amounts can influence meat texture parameters. The prooxidant effects of phytochemicals abundant in EOs lead to crosslinking through Schiff base formation, resulting in changes in primary texture attributes due to protein carbonylation [26]. The connective tissue changes depend on the soluble and total collagen fractions, and collagen changes are described to be responsible for many meat texture changes. However, these changes depend on collagen maturity and exogenous factors, such as heating rate, moisture level, and cooking constraints [27]. The higher the collagen solubility, the greater the meat tenderness, with lower thermal stability [27]. Fiber type, collagen content, and solubility are factors that determine meat tenderness and can be influenced by pre-slaughter feeding practices. The proportion of collagen types I and III can also affect shear force. Sarcomere length is a characteristic influenced by muscle location, carcass chilling rate, proteolysis rate, rigor mortis stage, and body weight at slaughter [28].

**Table 3.** Mean TBA values of untreated and treated on smoked meat (Se’i sapi) samples during storage period at room temperature (Mean ± SE).

Treatment	Days			
	0	7	14	21
Control	0.045 ± 0.00^a^	0.12 ± 0.00^c^	0.13 ± 0.01^e^	0.19 ± 0.00^d^
C 1 (0.1%)	0.049 ± 0.00^a^	0.12 ± 0.01^c^	0.13 ± 0.01^e^	0.13 ± 0.00^b^
C 2 (0.3 %)	0.096 ± 0.01^c^	0.09 ± 0.00^ab^	0.10 ± 0.01^cd^	0.11 ± 0.01^a^
C 3 (0.5%)	0.074 ± 0.00^b^	0.09 ± 0.00^ab^	0.09 ± 0.01^b^	0.10 ± 0.00^a^
L 1 (0.1%)	0.048 ± 0.00^a^	0.11 ± 0.00^bc^	0.13 ± 0.00^e^	0.15 ± 0.02^c^
L 2 (0.3%)	0.048 ± 0.00^a^	0.09 ± 0.02^b^	0.11 ± 0.00^de^	0.15 ± 0.00^c^
L 3 (0.5%)	0.046 ± 0.00^a^	0.08 ± 0.01^a^	0.08 ± 0.01^a^	0.09 ± 0.00^a^

Results are expressed as mean ± SD.

^abcd^ Significantly different in same column (additional EOs effect) (*p *< 0.05)

### TBA values

Based on the recent results presented in Table 3, the TBA values for the control sample were 0.045, 0.12, 0.13, and 0.19 on days 0, 7, 14, and 21 of storage, respectively. When using cinnamon bark oil, the TBA values for smoked meat (Se’i sapi) at 0.1% concentration (C1) were 0.049, 0.12, 0.13, and 0.13; at 0.3% concentration (C2), the TBA values were 0.096, 0.09, 0.10, and 0.11; and at 0.5% concentration (C3), the TBA values were 0.074, 0.09, 0.09, and 0.10 on the corresponding storage days. Using lime leaf oil, the TBA values for smoked meat (Se’i sapi) at 0.1% concentration (L1) were 0.048, 0.11, 0.13, and 0.15; at 0.3% concentration (L2), the TBA values were 0.048, 0.11, 0.13, and 0.15; and at 0.5% concentration (L3), the TBA values were 0.046, 0.08, 0.08, and 0.09 on the corresponding storage days.

**Figure 3.**
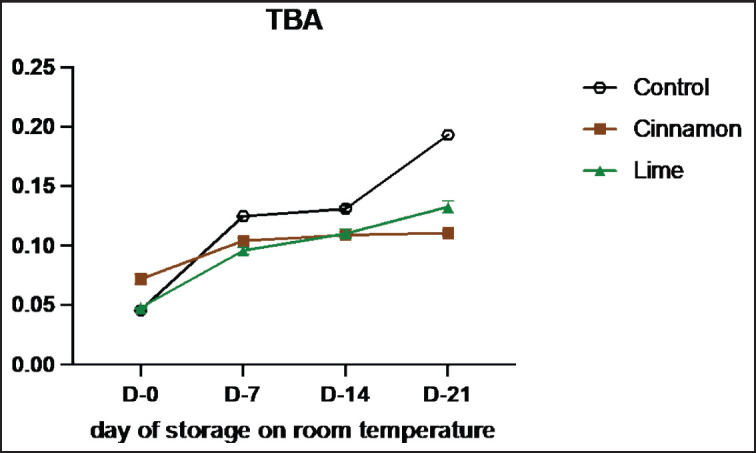
Average results of the addition of Cinnamons and Lime leaf EOs on the change in TBAs values during 21 days at room temperature.

The TBA values in smoked meat treated with cinnamon bark oil and lime leaf oil were lower than those in the control sample. Increasing the treatment concentration (using EOs) resulted in decreased TBA values (Fig. 3). These findings align with a previous study, which suggests that lower TBA values may be attributed to the high antioxidant effects [29]. Cinnamon bark oil contains polyphenolic compounds such as flavonoids, tannins, proanthocyanidins, and coumarin [30], as well as the active compound eugenol, which acts as a natural antioxidant [31].

Lime leaf oil also contains various active compounds, including flavonoids like quercetin and phenolics, which exhibit antioxidant properties. Lime leaf is rich in vitamin C, vitamin A, sulfur, citric acid, glycosides, dammar, and EOs. Additionally, lime leaf contains saponins and flavonoids such as hesperidin, tangeritin, naringin, eriocitrin, and eriocitrocide [15]. Interestingly, at specific concentrations, cinnamon bark oil resulted in higher TBA values compared to lime leaf oil. This suggests that lime leaf oil has a more effective impact in inhibiting lipid oxidation in meat. It can be inferred that the antioxidant properties of lime leaf oil are stronger than those of cinnamon bark oil due to the presence of compounds like quercetin, which is a potent antioxidant.

### Color analysis

**Figure 4.**
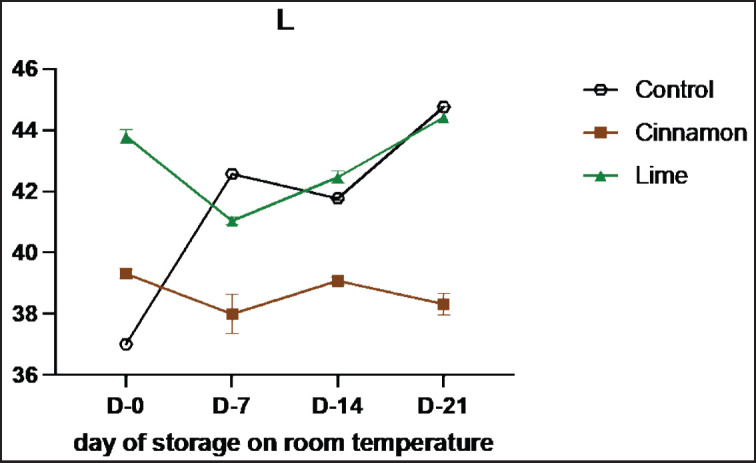
Average results of the addition of Cinnamons and Lime leaf EOs on the change in L values during 21 days at room temperature.

The results of the color parameters in Se’i sapi added with EOs were observed for the levels of brightness—L*, redness—a*, yellowness—b* during room temperature. It was reported that there was an increase in the L* value in the control from 37.02 on day 0 to 44.77 on day 21 (Fig. 4 and Table 4). The result of Se’i sapi added with lime leaf oils also experienced an increase in the L* value, from 43.29 on day 0 to 44.62 on day 21. Meanwhile, the addition of cinnamon essential oil experienced a decrease in the L* value from 39.3 on day 0 to 38.3 on day 21.

**Figure 5.**
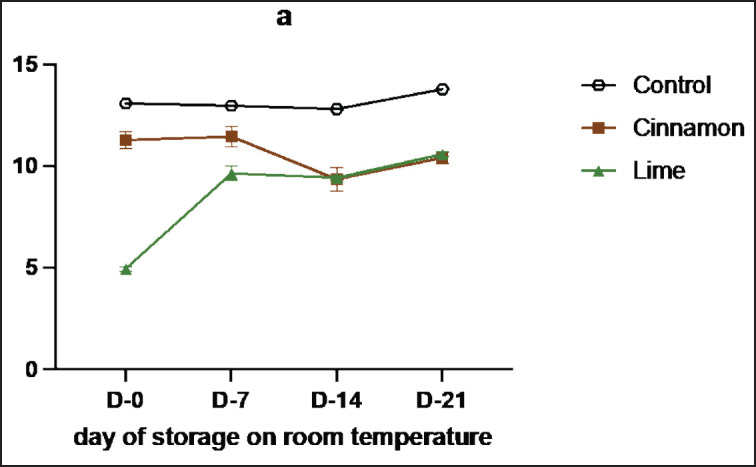
Average results of the addition of Cinnamons and Lime leaf EOs on the change in a values during 21 days at room temperature.

Color can be the first indicator of lipid degradation in meat and meat products. Color changes occur due to the oxidation of myoglobin to oxymyoglobin and metmyoglobin, which produces brown pigments that cause poor appearance of meat or meat products [18]. In addition, the instability of myoglobin during storage causes damage during freezing [19]. The redness value—a* in the control and Se’i sapi added with lime leaf oils increased (Fig. 5). The a* value of the control on day 0 was 13.09 and 13.78 on day 21. Meanwhile, for Se’i sapi which added lime leaf essential oil, the a* value on day 0 was 4.95 to 10.59 on day 21 (Table 5). For the a* parameter in Se’i sapi added with cinnamon essential oil, it decreased from 11.6 on day 0 to 10.4 on day 21. Sausages added with EOs coriander showed a decrease in color, especially brightness (L*), in treated samples; this phenomenon is caused by the interaction between myoglobin and the bioactive compound coriander EOs. Likely, this interaction is also responsible for the decrease in a* during the storage of Se’i sapi [20].

**Figure 6.**
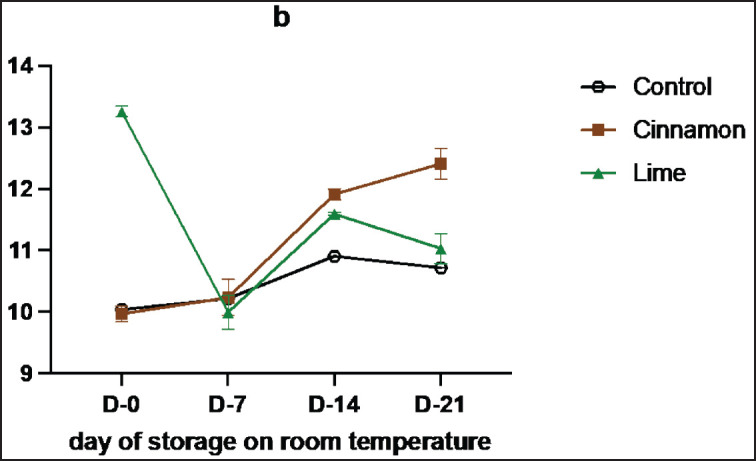
Average results of the addition of Cinnamons and Lime leaf EOs on the change in b values during 21 days at room temperature.

The yellowness parameter—b*—in the control sample, there was no difference (Fig. 6). On the other hand, in the sample added with cinnamon essential oil, it increased; on day 0, it was 9.96 to 12.41 on day 21, while in the sample added with lime leaf essential oil, it decreased from 13.25 on day 0 to 11.02 on day 21 (Table 6). The last research, which used oregano essential oil, also reported changes on the surface of the meat. An increase in the b* value indicates an increase in the yellow/brownish color of the meat; it can be assumed that the addition of the essential oil used affects the color of the meat [32].

**Table 4.** Mean L values of untreated and treated on smoked meat (Se’i sapi) samples during storage period at room temperature (Mean ± SE).

Treatment	Days			
	0	7	14	21
Control	37.02 ± 0.04^a^	42.57 ± 0.01^g^	41.77 ± 0.03^e^	44.77 ± 0.01^f^
C 1 (0.1%)	38.02 ± 0.03^b^	38.59 ± 0.06^b^	39.83 ± 0.01^c^	37.20 ± 0.02^b^
C 2 (0.3 %)	39.55 ± 0.01^c^	42.25 ± 0.07^f^	38.14 ± 0.01^a^	41.13 ± 0.01^c^
C 3 (0.5%)	40.37 ± 0.02^d^	33.15 ± 0.08^a^	39.27 ± 0.01^b^	36.62 ± 0.01^a^
L 1 (0.1%)	43.23 ± 0.01^f^	42.07 ± 0.02e	42.60 ± 0.01^f^	41.77 ± 0.03^d^
L 2 (0.3%)	44.87 ± 0.01^g^	40.61 ± 0.41^c^	40.99 ± 0.09^d^	47.68 ± 0.01^g^
L 3 (0.5%)	41.79 ± 0.02^e^	40.61 ± 0.02^d^	43.81 ± 0.02^g^	44.42 ± 0.01^e^

Results are expressed as mean ± SD.

^abcd^ Significantly different in same column (additional EOs effect) (*p *< 0.05)

**Table 5.** Mean a values of untreated and treated on smoked meat (Se’i sapi) samples during storage period at room temperature (Mean ± SE).

Treatment	Days			
	0	7	14	21
Control	13.09 ± 0.04^ f^	12.97 ± 0.01^ f^	12.82 ± 0.01^ f^	13.78 ± 0.01^ g^
C 1 (0.1%)	12.87 ± 0.04^ e^	14.64 ± 0.02^ g^	7.09 ± 0.02^ b^	9.79 ± 0.01^ c^
C 2 (0.3 %)	13.17 ± 0.01^ g^	12.25 ± 0.03^ de^	14.03 ± 0.01^ g^	12.58 ± 0.23^ f^
C 3 (0.5%)	8.79 ± 0.02^ d^	7.51 ± 0.27^ b^	6.93 ± 3.42^ a^	8.85 ± 0.02^ a^
L 1 (0.1%)	4.34 ± 0.07^ a^	12.23 ± 0.01^ de^	10.52 ± 0.02^ e^	10.67 ± 0.03^ d^
L 2 (0.3%)	5.87 ± 0.05^ c^	9.61 ± 0.12^ c^	8.89 ± 0.02^ cd^	9.52 ± 0.02^ b^
L 3 (0.5%)	4.64 ± 0.03^ b^	7.08 ± 0.16^ a^	8.89 ± 0.02^ cd^	11.59 ± 0.02^ e^

Results are expressed as mean ± SD.

^abcd^ Significantly different in same column (additional EOs effect) (*p *< 0.05)

### Sensory evaluation

**Figure 7.**
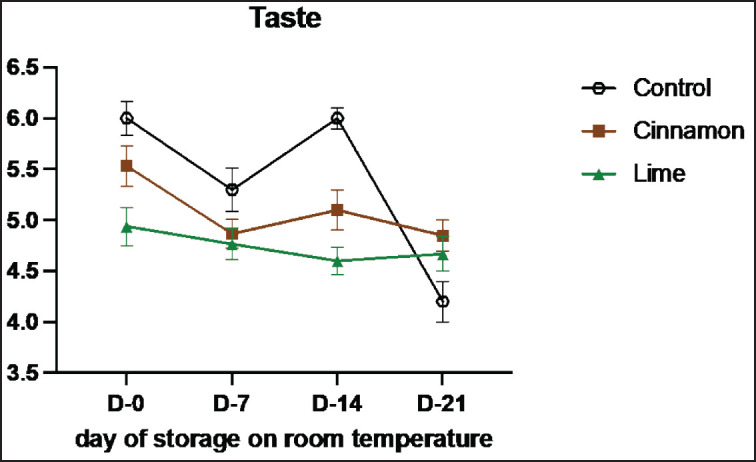
Average results of the addition of Cinnamons and Lime leaf EOs on the change in taste values on sensory evaluation during 21 days at room temperature.

**Figure 8.**
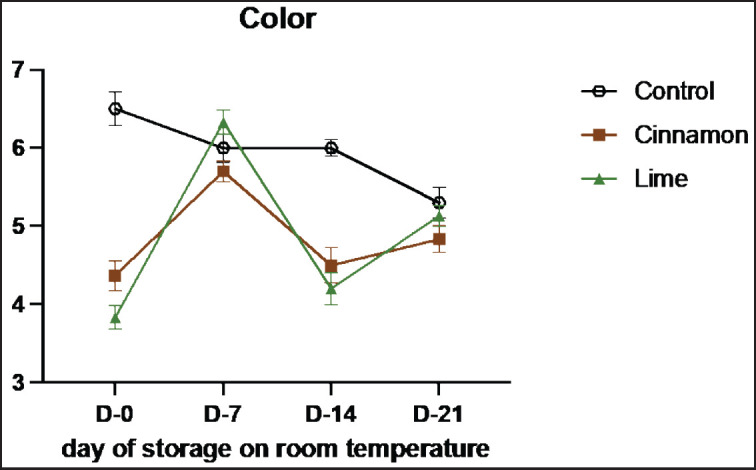
Average results of the addition of Cinnamons and Lime leaf EOs on the change in color values on sensory evaluation during 21 days at room temperature.

**Figure 9.**
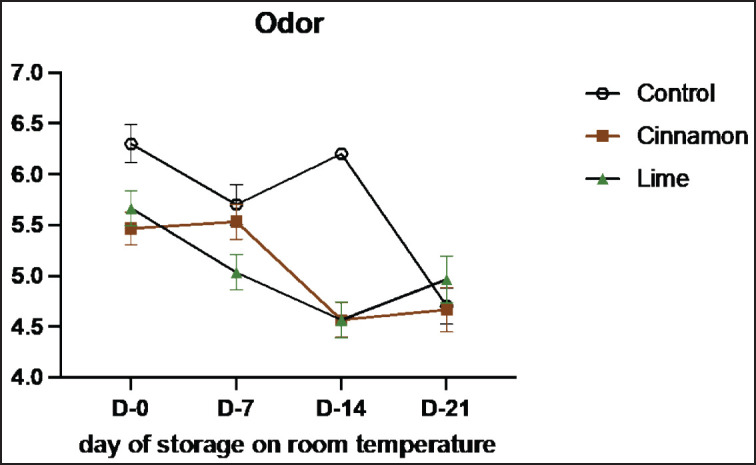
Average results of the addition of Cinnamons and Lime leaf EOs on the change in odor values on sensory evaluation during 21 days at room temperature.

**Figure 10.**
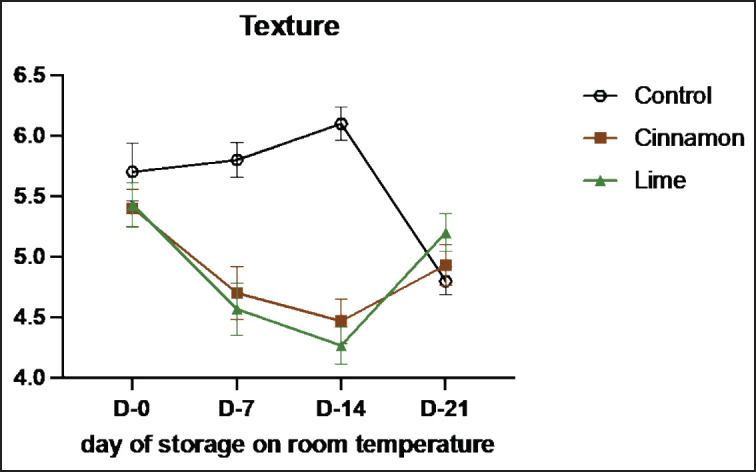
Average results of the addition of Cinnamons and Lime leaf EOs on the change in texture values on sensory evaluation during 21 days at room temperature.

**Figure 11.**
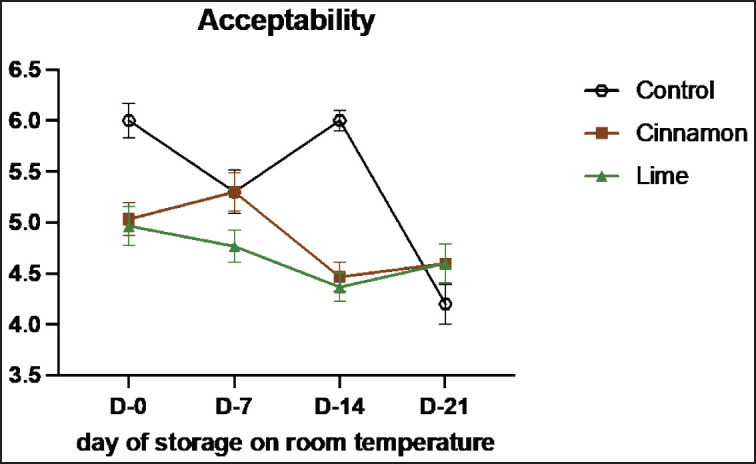
Average results of the addition of Cinnamons and Lime leaf EOs on the change in acceptability values on sensory evaluation during 21 days at room temperature.

Sensory evaluation is a vital method for assessing the quality and characteristics of food products through human senses, such as vision, smell, taste, and texture [33]. This study highlights the role of cinnamon (C) and lime leaf (L) EOs in enhancing the sensory attributes and shelf life of smoked beef (se’i sapi) during room storage (Tables 7–11). Both treatments showed remarkable improvements in sensory qualities, with the most notable results observed on Day 14. Cinnamon significantly enhanced taste (from 3.55 on Day 0 to 4.36 on Day 14), texture (4.45 to 7.00), and overall acceptance (3.82 to 4.36), demonstrating its ability to retain moisture, stabilize structure, and improve flavor. Similarly, Lime improved texture and acceptance, although its aromatic compounds displayed higher volatility, peaking at 5.55 on Day 7 and slightly declining to 5.09 by Day 14. By Day 21, both treatments experienced minor declines, likely due to natural oxidative changes and compound degradation, with cinnamon showing better stability than lime leaf. These results show that EOs can improve the taste of food and keep it fresh naturally by stopping lipid oxidation and microbial growth (Figs. 7-11) [34].

**Table 6.** Mean b values of untreated and treated on smoked meat (Se’i sapi) samples during storage period at room temperature (Mean ± SE).

Treatment	Days			
	0	7	14	21
Control	10.04 ± 0.014^ b^	10.22 ± 0.01^ cd^	10.91 ± 0.02^ a^	10.72 ± 0.01^ bc^
C 1 (0.1%)	10.266 ± 0.01^ c^	10.54 ± 0.04^ cde^	12.59 ± 0.01^ g^	14.27 ± 0.04^ g^
C 2 (0.3 %)	10.67 ± 0.03^ d^	12.12 ± 0.06^ g^	11.34 ± 0.02^ b^	12.25 ± 0.03^ f^
C 3 (0.5%)	8.97 ± 0.03^ a^	8.04 ± 0.02^ ab^	11.72 ± 0.01^ f^	10.73 ± 0.02^ bc^
L 1 (0.1%)	13.05 ± 0.05^ f^	11.14 ± 1.07^ ef^	11.86 ± 0.03^ c^	11.86 ± 0.04^ d^
L 2 (0.3%)	13.95 ± 0.04^ g^	7.88 ± 0.17^ ab^	11.68 ± 0.04^ de^	9.06 ± 0.01^ a^
L 3 (0.5%)	12.75 ± 0.05^ e^	10.97 ± 0.03^ ef^	11.67 ± 0.04^ de^	12.16 ± 0.01^ e^

Results are expressed as mean ± SD.

^abcd^ Significantly different in same column (additional EOs effect) (*p *< 0.05)

**Table 7.** Mean the taste of organoleptic values of untreated and treated on smoked meat (Se’i sapi) samples during storage period at room temperature (Mean ± SE).

Treatment	Days			
	0	7	14	21
Control	6.00 ± 1.33	5.30 ± 1.70	6.00 ± 0.82^b^	4.20 ± 1.55
C1 (0.1%)	4.20 ± 1.75	4.50 ± 1.18	5.30 ± 1.89^ab^	4.80 ± 1.32
C2 (0.3%)	4.20 ± 1.55	4.70 ± 0.95	5.20 ± 1.32^ab^	5.10 ± 1.29
C3 (0.5%)	5.20 ± 1.40	5.40 ± 1.26	4.20 ± 1.40^a^	5.40 ± 1.07
L1 (0.1%)	5.40 ± 1.43	5.10 ± 1.10	4.70 ± 1.25^ab^	5.60 ± 0.52
L2 (0.3%)	4.80 ± 1.75	4.60 ± 0.84	4.30 ± 1.16^a^	4.10 ± 1.45
L3 (0.3%)	4.70 ± 1.42	4.60 ± 1.71	4.10 ± 0.88^a^	4.10 ± 1.45

Results are expressed as mean ± SD.

^abcd^ Significantly different in same column (additional EOs effect) (*p* < 0.05)

**Table 8.** Mean color values of untreated and treated on smoked meat (Se’i sapi) samples during storage period at room temperature (Mean ± SE).

Treatment	Days			
	0	7	14	21
Control	6.50 ± 1.72^b^	6.00 ± 1.41	6.00 ± 0.82^b^	5.30 ± 1.57
C1 (0.1%)	4.20 ± 1.48^a^	5.90 ± 1.20	3.40 ± 1.65^a^	4.10 ± 1.45
C2 (0.3%)	5.20 ± 1.40^ab^	5.30 ± 0.95	5.90 ± 0.88^b^	5.50 ± 1.18
C3 (0.5%)	3.70 ± 1.42^a^	5.90 ± 1.10	4.20 ± 1.75^ab^	4.90 ± 1.20
L1 (0.1%)	3.60 ± 1.08^a^	6.40 ± 1.71	5.00 ± 1.05^ab^	5.20 ± 1.14
L2 (0.3%)	4.00 ± 1.41^a^	6.30 ± 0.95	3.30 ± 1.77^a^	5.20 ± 1.14
L3 (0.3%)	6.50 ± 1.20^a^	6.30 ± 1.25	4.30 ± 1.57^ab^	5.00 ± 1.25

Results are expressed as mean ± SD.

^abcd^ Significantly different in same column (additional EOs effect) (*p *< 0.05)

**Table 9.** Mean odor values of untreated and treated on smoked meat (Se’i sapi) samples during storage period at room temperature (Mean ± SE).

Treatment	Days			
	0	7	14	21
Control	6.30 ± 1.49	5.70 ± 1.57	6.20 ± 0.42	4.70 ± 1.42
C1 (0.1%)	5.60 ± 1.35	5.20 ± 1.23	4.40 ± 1.35	4.90 ± 2.02
C2 (0.3%)	5.20 ± 1.23	5.80 ± 1.40	4.90 ± 1.29	4.60 ± 1.51
C3 (0.5%)	5.60 ± 1.43	5.60 ± 1.58	4.40 ± 1.51	4.50 ± 1.84
L1 (0.1%)	5.70 ± 0.95	5.60 ± 1.35	4.90 ± 1.45	4.10 ± 1.66
L2 (0.3%)	5.60 ± 1.51	5.10 ± 1.37	4.40 ± 1.58	5.50 ± 2.07
L3 (0.3%)	5.70 ± 1.70	4.40 ± 1.26	4.40 ± 1.35	5.30 ± 1.57

Results are expressed as mean ± SD.

^abcd^ Significantly different in same column (additional EOs effect) (*p* < 0.05)

**Table 10.** Mean texture values of untreated and treated on smoked meat (Se’i sapi) samples during storage period at room temperature (Mean ± SE).

Treatment	Days			
	0	7	14	21
Control	5.70 ± 1.89	5.80 ± 1.14^ab^	6.10 ± 1.10^b^	4.80 ± 0.92
C1 (0.1%)	5.40 ± 1.35	5.50 ± 1.18^ab^	3.90 ± 0.88^a^	4.90 ± 1.29
C2 (0.3%)	5.60 ± 1.08	4.50 ± 1.65^ab^	5.80 ± 0.79^a^	5.50 ± 1.35
C3 (0.5%)	5.20 ± 1.40	4.10 ± 2.13^ab^	3.40 ± 1.43^a^	4.40 ± 1.35
L1 (0.1%)	5.50 ± 1.51	4.00 ± 1.76^ab^	4.50 ± 1.08^a^	5.90 ± 1.37
L2 (0.3%)	5.60 ± 1.71	3.80 ± 1.40^a^	3.90 ± 1.20^a^	5.10 ± 1.10
L3 (0.3%)	5.20 ± 1.14	5.90 ± 1.20^b^	4.40 ± 1.35^a^	4.60 ± 0.97

Results are expressed as mean ± SD.

^abcd^ Significantly different in same column (additional EOs effect) (*p* < 0.05)

**Table 11.** Mean acceptability values of untreated and treated on smoked meat (Se’i sapi) samples during storage period at room temperature (Mean ± SE).

Treatment	Days			
	0	7	14	21
Control	6.00 ± 1.33	5.30 ± 1.70	6.00 ± 0.87^b^	4.20 ± 1.55
C1 (0.1%)	4.80 ± 1.32	5.30 ± 1.88	4.50 ± 1.18^a^	4.20 ± 1.75
C2 (0.3%)	5.10 ± 1.29	5.20 ± 1.32	4.70 ± 0.95^ab^	4.20 ± 1.55
C3 (0.5%)	5.20 ± 1.40	5.40 ± 1.26	4.20 ± 1.40^a^	5.40 ± 1.07
L1 (0.1%)	5.40 ± 1.43	5.10 ± 1.10	4.70 ± 1.25^ab^	5.60 ± 0.52
L2 (0.3%)	4.80 ± 1.75	4.60 ± 0.84	4.30 ± 1.16^a^	4.10 ± 1.45
L3 (0.3%)	4.70 ± 1.41	4.60 ± 1.71	4.10 ± 0.87^a^	4.10 ± 1.44

Results are expressed as mean ± SD.

^abcd^ Significantly different in same column (additional EOs effect) (*p *< 0.05)

The study not only supports the idea that these oils could change the way food is stored, but it also helps the food industry come up with healthier, more environmentally friendly alternatives. The result meets the growing need around the world for natural and useful food additives.

## Conclusion

The addition of lime leaf and cinnamon EOs affected the pH, TBA values, color, and sensory parameters of the smoked meat (Se’i sapi). However, no significant difference in tenderness was found between the treatments. The addition of lime leaf and cinnamon EOs to Se’i sapi lowered the pH and TBA values of the smoked meat during room temperature storage compared to the control samples, particularly with the addition of 0.5% cinnamon EOs and lime leaf EOs on day 21. The addition of cinnamon essential oil decreased the brightness (L*) and redness (a*), while the addition of lime leaf essential oil increased the brightness (L*) and redness (a*). Furthermore, the addition of both EOs at 0.5% maintained the meat product quality in Se’i sapi better than other treatments after 21 days of storage, due to their antibacterial and antioxidant properties.

## List of abbreviations

a*, redness; b*, yellowness; C, celsius; cm, centimeter; CRD, completely randomized design; EOs, essential oils; GRAS, generally recognized as safe; h, hours; HCL, hydrogen chlorides; L*, lightness; ml, milliliter; min, minutes; nm, nanometers; PP, polypropylene; TBA, thiobarbituric acid; RPM, revolutions per minute.
